# Depression in systemic lupus erythematosus: Modifiable or inheritable? a two-sample mendelian randomization study

**DOI:** 10.3389/fgene.2022.988022

**Published:** 2022-08-30

**Authors:** Jinyun Chen, Ting Xu, Min Wu

**Affiliations:** Department of Rheumatology and Immunology, Third Affiliated Hospital of Soochow University, Changzhou, China

**Keywords:** depression, systemic lupus erythematosus, mendelian randomization, causality, genetic association

## Abstract

Observational studies have found increased incidence of depression, the leading cause of disability worldwide, in patients with systemic lupus erythematosus (SLE). However, it is not clear whether the association was genetically inheritable or caused by modifiable risk factors, such as socioeconomic factors. We investigated the causal relationship between genetically predicted SLE and depression by two-sample Mendelian randomization analysis. Single nucleotide polymorphisms (SNPs) associated with SLE were selected as instrumental variables (IVs) from a genome-wide association study (GWAS) of 14,267 European-ancestry participants. A large GWAS of depression (180,866 European-ancestry participants) and another GWAS of major depressive disorder (MDD) (173,005 European-ancestry participants) were selected as outcomes. Then we estimated the effects of IVs on the odds of depression or MDD by using the inverse-variance weighted (IVW) meta-analysis method (random-effects), which had a power of 90% to detect 4% increase of depression in SLE. Interestingly, genetically predicted SLE decreased the odds of depression [odds ratio (OR): 0.995; 95% CI: 0.990–0.999; *p* = 0.025] and MDD [odds ratio (OR): 0.985; 95% CI: 0.975–0.996; *p* = 0.009], indicating increased depression in SLE was not due to inheritable risk factors.

## Introduction

Systemic lupus erythematosus (SLE) is a chronic autoimmune disease that may influence multiple organs, including central nervous system (CNS) involvement. A recent meta-analysis showed a pooled prevalence of 35.0% of depression in SLE patients (Moustafa et al., 2020), which was much higher than that in the general population (Bachen et al., 2009). Risk factors for depression can be divided into two categories: unmodifiable and modifiable. The former include genetic vulnerability and early-life adversity (Sullivan et al., 2000; Köhler et al., 2018), while the latter include socioeconomic (e.g., activities, family support, education, stress), behavioral (e.g., exercise, sleep, diet), and environmental (e.g., noise pollution) variables (Choi et al., 2020). It has been reported that depression in SLE was associated with both disease-related factors [e.g., pain and fatigue (Monahan et al., 2021), increased disease activity (Knight et al., 2018), musculoskeletal and skin system involvement (Eldeiry et al., 2020)] and modifiable factors [e.g., lower education (Knight et al., 2018), financial strain (Mccormick et al., 2018), physical inactivity (Patterson et al., 2021), relationship satisfaction (Figueiredo-Braga et al., 2018)]. However, we do not know which factors play a crucial and major role, for there may be crosstalk and potential confounders among them. Thus, we conducted two-sample Mendelian randomization (MR) analysis to elucidate the causal relationship between genetically predicted SLE and depression.

## Materials and methods

### Study populations

The MR analysis included the largest GWAS meta-analysis on SLE (*n* = 14,267) as exposure, and a large GWAS of depression (*n* = 180,866) and another GWAS of major depressive disorder (MDD) (*n* = 173,005) as outcomes, for whom summary-level data were available (Bentham et al., 2015; Okbay et al., 2016; Wray et al., 2018). All of the participants were of European ancestry and provided written informed consent in each of the contributing studies. We acquired summary data for all SNPs by our search from MR Base database (Hemani et al., 2018).

### Instrumental variables

We searched the MR Base database on 8 June 2022, to identify SNPs associated with SLE (using a *p*-value threshold of 5 × 10^–8^), and exclude those that were in linkage disequilibrium (within 10000 kb or r^2^ > 0.001). There were a total of 43 independent SNPs associated with SLE, 31 and 40 of which were selected as instrumental variables (IVs) against depression and MDD, respectively (Supplementary Table S1). Then we applied 2-sample MR to estimate the effect of SLE on depression or MDD.

### Statistical analysis

Inference of causality in the estimated etiological associations between SLE and depression or MDD depends on the satisfaction of MR assumptions (Haycock et al., 2016): 1) the selected SNPs are associated with SLE; 2) the selected SNPs are not associated with confounders; and 3) the selected SNPs are associated with depression or MDD exclusively through their effect on SLE. If the above assumptions are satisfied, the selected SNPs are valid IVs, and their association with depression or MDD can be interpreted as a causal effect of SLE.

The first assumption can be satisfied by restricting the IVs to SNPs that were discovered using genome-wide statistical significance (*p* ≤ 5 × 10^–8^) and replicated in independent studies. The other two assumptions are unprovable, and, when violated, can lead to bias in MR analyses. However, they can be investigated by estimating the relation between the IVs and a wide range of characteristics. Violations of the third assumption can be introduced by horizontal pleiotropy. If there is no horizontal pleiotropy, or the horizontal pleiotropy is balanced (no directional horizontal pleiotropy), an unbiased causal estimate can be obtained by inverse-variance weighted (IVW) linear regression. MR-Egger regression can provide valid causal estimates even in the presence of horizontal pleiotropy, though less precise than other methods.

The association between genetically predicted SLE and depression or MDD attributable to each SNP was accessed with the Wald method, which computes the ratio between the SNP-SLE and SNP-depression or SNP-MDD estimates. The ratio estimates for individual SNPs were combined by using the IVW meta-analysis method (random-effects). We additionally examined the violation of the MR assumptions through sensitivity analyses, based on the weighted median, MR-Egger, and weighted mode approaches. Horizontal pleiotropy was examined from MR-Egger intercept test. Cochran’s Q value was furthermore calculated for the IVW and MR-Egger estimates to measure the heterogeneity. If there was significant heterogeneity between SNPs, they would be further analyzed with outlier-corrected MR-PRESSO (Verbanck et al., 2018).

All the above analyses were performed in R, version 3.5.1. *p* values were 2-sided, and evidence of association was declared at *p* < 0.05. Power of the MR analysis was estimated with an online calculator by Burgess et al. at https://sb452.shinyapps.io/power/.

## Results

The identified risk loci in the GWAS for SLE explained an estimated 19.3% of the total genetic susceptibility to SLE (Bentham et al., 2015). We selected 31 and 40 SNPs from the GWAS as IVs for SLE risk against depression and MDD, respectively, with both F-statistics >30000. The selected SNPs correspond to independent genomic regions with an odds ratio (OR) from 1.18 to 2.53.

**FIGURE 1:**
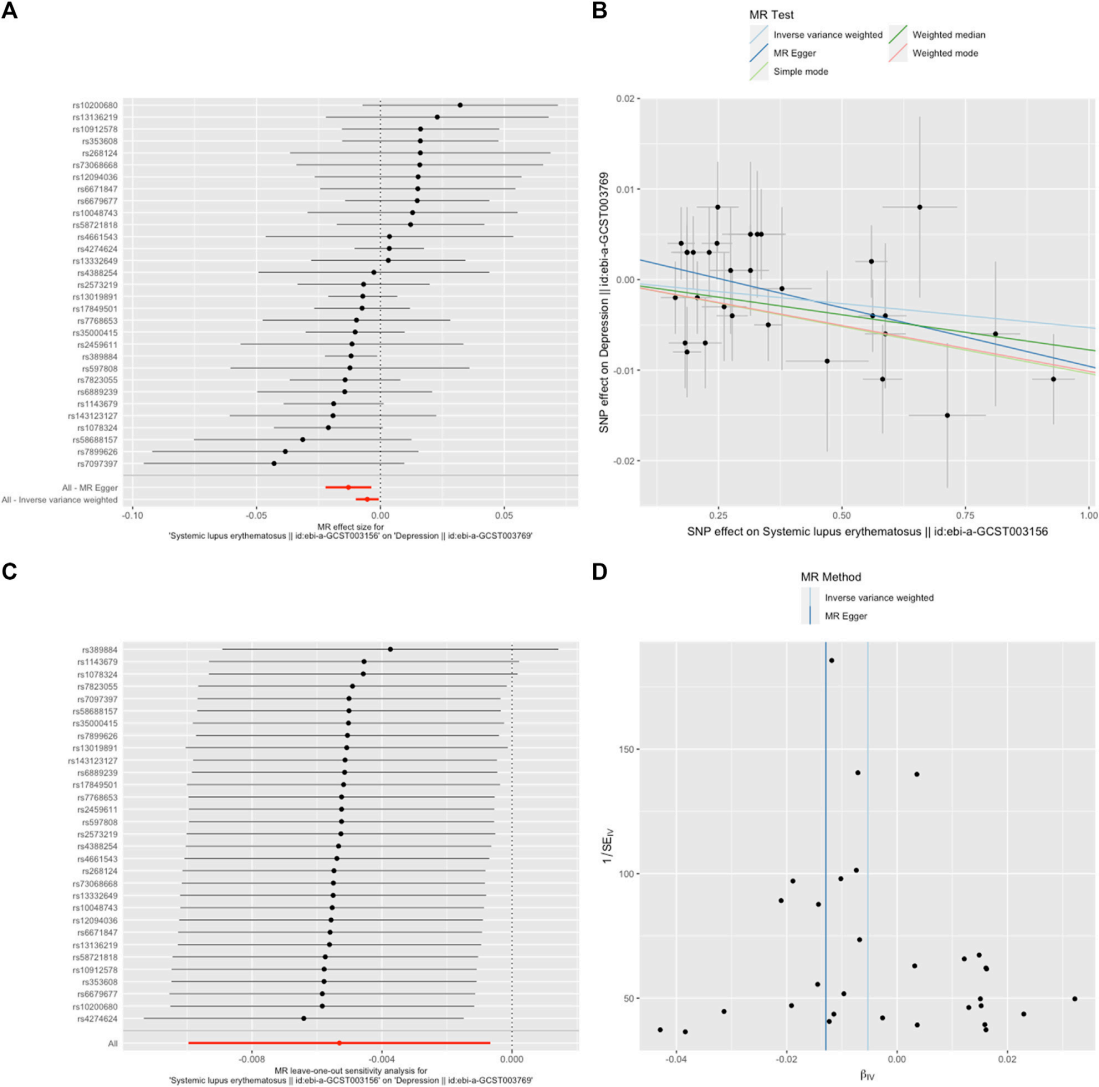
SLE decreased the odds of depression. **(A)**. The forest plot shows the estimate of the effect of genetically increased SLE risk on depression risk, where each black point represents the log odds ratio (OR) for depression per standard deviation (SD) increase in log OR for SLE, and red points showing the combined causal estimate using all SNPs together in a single instrument, using each of the two different methods (inverse-variance weighted [IVW] random effects and MR-Egger). Horizontal lines denote 95% confidence intervals (95%CIs). **(B)**. A plot relating the effect sizes of the SNP-SLE association (x-axis, log OR) and the SNP-depression associations (y-axis, log OR) with standard error bars. The slopes of the lines correspond to causal estimates using each of the four different methods (weighted median, weighted mode, IVW random effects and MR-Egger) **(C)**. MR leave-one-out sensitivity analysis for SLE on depression. Each black point represents the IVW MR method applied to estimate the causal effect of SLE on depression excluding that particular variant from the analysis. The red point depicts the IVW estimate using all SNPs. There are no instances where the exclusion of one particular SNP leads to dramatic changes in the overall result. **(D)**. Funnel plot showing the relationship between the causal effect of SLE on depression estimated using each individual SNP as a separate instrument against the inverse of the standard error of the causal estimate. Vertical lines show the causal estimates using all SNPs combined into a single instrument for each of the two different methods (IVW random effects and MR-Egger). There is no significant asymmetry in the plot.

**FIGURE 2:**
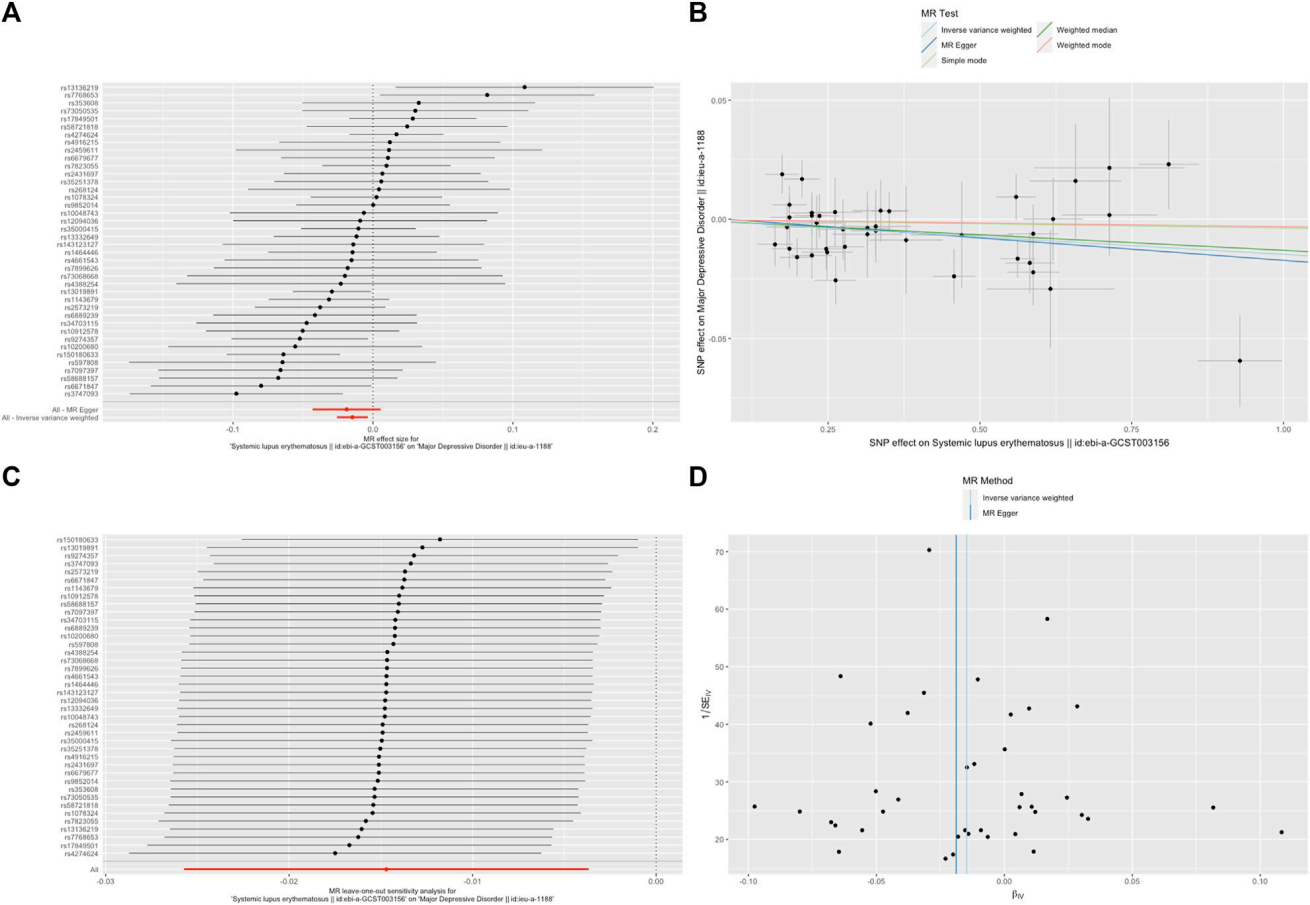
SLE decreased the odds of major depressive disorder (MDD). **(A)**. The forest plot shows the estimate of the effect of genetically increased SLE risk on MDD risk, where each black point represents the log odds ratio (OR) for MDD per standard deviation (SD) increase in log OR for SLE, and red points showing the combined causal estimate using all SNPs together in a single instrument, using each of the two different methods (inverse-variance weighted [IVW] random effects and MR-Egger). Horizontal lines denote 95% confidence intervals (95%CIs). **(B)**. A plot relating the effect sizes of the SNP-SLE association (x-axis, log OR) and the SNP-MDD associations (y-axis, log OR) with standard error bars. The slopes of the lines correspond to causal estimates using each of the four different methods (weighted median, weighted mode, IVW random effects and MR-Egger). **(C)**. MR leave-one-out sensitivity analysis for SLE on MDD. Each black point represents the IVW MR method applied to estimate the causal effect of SLE on MDD excluding that particular variant from the analysis. The red point depicts the IVW estimate using all SNPs. There are no instances where the exclusion of one particular SNP leads to dramatic changes in the overall result. **(D)**. Funnel plot showing the relationship between the causal effect of SLE on MDD estimated using each individual SNP as a separate instrument against the inverse of the standard error of the causal estimate. Vertical lines show the causal estimates using all SNPs combined into a single instrument for each of the two different methods (IVW random effects and MR-Egger). There is no significant asymmetry in the plot.

A random-effects IVW model yielded a pooled MR estimate of significant effect, demonstrating that SLE mildly decreased the odds of depression by 0.995 (95% CI: 0.990–0.999; *p* = 0.025) (Figure 1) and MDD by 0.985 (95% CI: 0.975–0.996; *p* = 0.009) (Figure 2). Weighted median and weighted mode estimates for the candidate SNPs yielded results similar to those of the random-effects IVW model (Table 1). There was no evidence of heterogeneity in the IVW and MR-Egger analysis (*p* > 0.05). And we did not find the presence of directional horizontal pleiotropy in the analysis, as indicated by the MR-Egger intercept test (*p* > 0.05). Further leave-one-out analysis, MR-PRESSO and funnel plot analysis didn’t detect significant outliers.

**TABLE 1:** Two-sample MR estimates for the effect of SLE on depression or MDD.

Outcomes	Method	OR (95%CI)	*p* value	Heterogeneity	Pleiotropy
Depression	MR Egger	0.987 (0.978–0.996)	0.011	>0.05	>0.05
	Weighted median	0.992 (0.986–0.999)	0.021		
	Inverse variance weighted	0.995 (0.990–0.999)	0.025	>0.05	
	Weighted mode	0.990 (0.983–0.997)	0.009		
	MR-PRESSO	0.995 (0.990–0.999)	0.025		
MDD	MR Egger	0.981 (0.958–1.006)	0.1381	>0.05	>0.05
	Weighted median	0.987 (0.972–1.002)	0.09151		
	Inverse variance weighted	0.985 (0.975–0.996)	0.00896	>0.05	
	Weighted mode	0.997 (0.967–1.027)	0.8396		
	MR-PRESSO	0.985 (0.975–0.996)	0.00896		

OR, odds ratio; Heterogeneity, *p* value for Cochran’s Q test; Pleiotropy, *p* value for MR-Egger intercept test.

## Discussion

Our study demonstrated for the first time that genetically predicted SLE might decrease the risk of depression. This finding gave us a new vision that increased depression in SLE may be more likely to be attributed to modifiable factors rather than inheritable factors.

Depression can occur for a variety of reasons, such as stress, personality, loneliness, family history, illness, and alcohol or drug use. The most reported SLE-specific factors including pain and fatigue (Monahan et al., 2021), increased disease activity (Knight et al., 2018), and musculoskeletal and skin system involvement (Elefante et al., 2022) (Eldeiry et al., 2020). Most of them could influence modifiable factors, such as self-esteem and strain, subsequently.

Nonetheless, a recent study found that SLE patients with depression/anxiety have increased blood brain barrier (BBB) permeability in the right insular area as compared with those without depression/anxiety (Wang et al., 2022). However, demographics and clinical features were not balanced between the two groups for small sample size. Another study showed accumulation of senescent neural cells in MRL/lpr SLE model mice with depressive behavior (Saito et al., 2021), which still needs to be verified in human.

Our study has some limitations. First, the participants in our analysis were all of European ancestry. Thus, our results should be applied cautiously to non-Europeans. Second, we included SNPs only those with a genome-wide significance level (*p* < 5 × 10^–8^), meaning those genuinely associated variants that did not reach the stringent *p*-value threshold were ignored. Third, though significantly, the effect size was very small. Despite this, our analyses were sufficiently powered (i.e., >90%) to detect as low as 4% increase of depression or MDD in SLE. And we utilized a range of methods as sensitivity analyses to test the robustness of the MR estimates against potential violations. Lastly, since it was impossible to prove MR assumptions 2) and 3), the direction of causality may be more reliable than the magnitude of causal effect, which was very sensitive to violations of assumptions.

## Conclusion

Genetically predicted SLE mildly decreased the risk of depression, indicating that increased depression in SLE was not inheritable. More investigations into the most relevant modifiable factors may help prevent depression in SLE patients.

## Data Availability

The datasets for this study can be found in the MR Base database, at http://app.mrbase.org.
